# Management of avian malaria in populations of high conservation concern

**DOI:** 10.1186/s13071-022-05327-2

**Published:** 2022-06-15

**Authors:** Andrea Miranda Paez, Kayleigh Chalkowski, Sarah Zohdy, Janna R. Willoughby

**Affiliations:** 1 College of Forestry, Wildlife and Environment, Auburn University, Auburn, AL USA; 2 College of Forestry, Wildlife and Environment and College of Veterinary Medicine, Auburn University, Auburn, AL USA

**Keywords:** Avian, Malaria, Mosquito, *Plasmodium*

## Abstract

Avian malaria is a vector-borne disease that is caused by *Plasmodium* parasites. These parasites are transmitted via mosquito bites and can cause sickness or death in a wide variety of birds, including many threatened and endangered species. This Primer first provides contextual background for the avian malaria system including the life cycle, geographic distribution and spread. Then, we focus on recent advances in understanding avian malaria ecology, including how avian malaria can lead to large ecosystem changes and variation in host immune responses to *Plasmodium* infection. Finally, we review advances in avian malaria management in vulnerable bird populations including genetic modification methods suitable for limiting the effects of this disease in wild populations and the use of sterile insect techniques to reduce vector abundance.

**Graphical Abstract:**

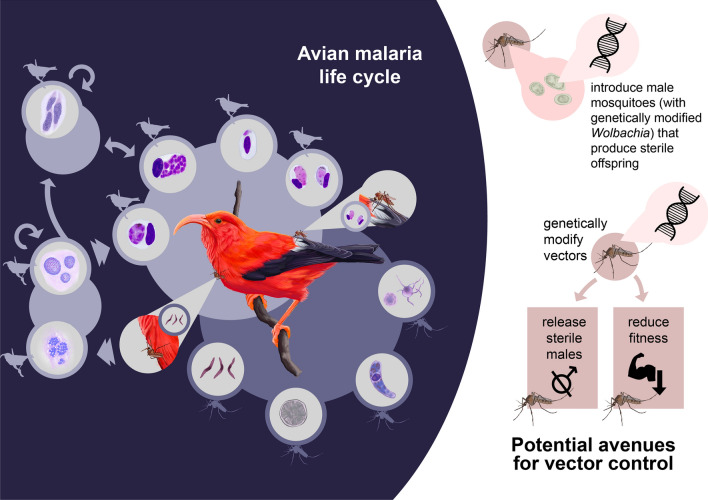

**Supplementary Information:**

The online version contains supplementary material available at 10.1186/s13071-022-05327-2.

## Transmission cycle of avian malaria

Avian malaria is a disease that infects various tissues and blood cells of birds and is caused by > 50 parasite species within the genus *Plasmodium* [1]. Malaria parasites require an invertebrate vector and a vertebrate host species to complete the life cycle. Vectors for *Plasmodium* include mosquitoes from the genera *Culex, Aedes* and *Culiseta*, and *Plasmodium* species are capable of infecting and completing their life cycle in > 400 species of birds, covering 11 orders [2]. The > 50 parasite species that cause avian malaria differ in characteristics such as host range, geographic distribution, competent vectors and pathogenicity [3]. Because the parasite's vectors and host species are so numerous and varied, the potential for avian malaria to negatively impact species as it spreads to new areas is high, resulting in the decline and even extinction of avian species in these newly invaded ecosystems.

**Fig. 1:**
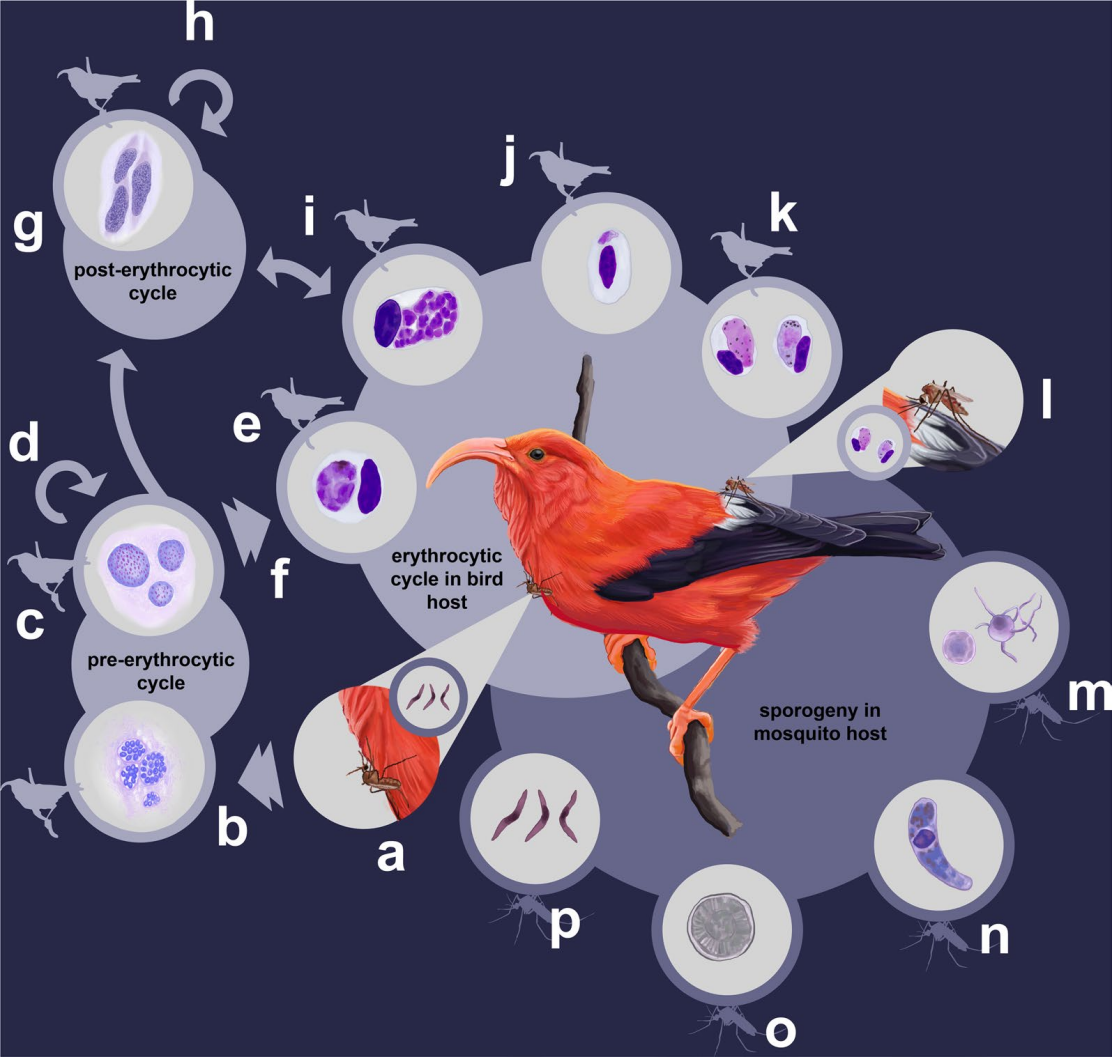
The avian malaria life cycle starts when (**a**) a feeding mosquito infects an avian host with *Plasmodium* sporozoites; (**b**) sporozoites then develop into exo-erythrocytic meronts (i.e. cryptozoites) in reticuloendothelial cells (e.g. spleen, liver, bone) throughout the body (**c**) followed by the development of merozoites into the second pre-erythrocytic exo-erythrytic stage and producing metacryptozoites. **d** Following development into metacryptozoites, further generations of this stage can be formed from previous generations. **e** Alternatively, merozoites from metacrytpzoites can enter the bloodstream, infect erythrocytes and (**f**) become meronts to continue into the erythritic cycle. **g** Merozoites can also develop into the next exo-erythrocytic form, the post-erythritic phanerozoites, which can (**h**) also develop further generations of phanerozoites or (**i**) develop into merozoites. Merozoites can be formed from either phanerozoites or erythrocytic meronts. From (**j**) merozoites, the erythrocytic cycle continues with the development of (**k**) male and female micro/macrogametocytes. These gametocytes are then (**l**) capable of infecting another mosquito to begin the process of sporogony in this next host. Once inside the mosquito, the gametocytes develop into (**m**) gametes in the midgut. These gametes come together to form (**n**) a zygote, which then develops into ookinetes that travel from the midgut to the epithelium, (**o**) followed by development into oocysts. Within the oocysts, (**p**) infective haploid sporozoites form and burst through the oocyst wall once reaching maturity. These haploid sporozoites then invade mosquito salivary glands, where they can be transferred to another bird host when the mosquito feeds

The avian malaria life cycle (Fig. 1) starts when a feeding mosquito infects an avian host with *Plasmodium* sporozoites, and sporozoites develop into exo-erythrocytic meronts (i.e. cryptozoites) in reticuloendothelial cells [4, 5]. This is followed by the development of merozoites into the second pre-erythrocytic exo-erythrytic stage, producing metacryptozoites. Further generations of metacryptozoites can be formed from previous generations, or alternatively merozoites from metacryptozoites can enter the bloodstream, infect erythrocytes and become meronts to continue into the erythritic cycle [5]. Merozoites can also develop into the next exo-erythrocytic form, the post-erythritic phanerozoites, which can develop into further generations of phanerozoites or develop into merozoites. From merozoites, the erythrocytic cycle continues with the development of male and female micro/macrogametocytes. These gametocytes are then capable of infecting another mosquito to begin the process of sporogony in this next host [5]. Once inside the mosquito, the gametocytes develop into gametes in the midgut. These gametes then come together to form a zygote, which develops into ookinetes that travel to the epithelium and develop into oocysts. Within the oocysts, infective haploid sporozoites form and, once mature, burst through the oocyst wall. These haploid sporozoites then invade mosquito salivary glands, where they can be transferred to another bird host when the mosquito feeds (Fig. 1).

## Distribution and spread of avian malaria

The native ranges of *Plasmodium* parasites are distributed worldwide, in diverse habitats (e.g. Nearctic, Palearctic, Oriental, Neotropical and Australian ecozones) [2], and co-vary with similarly wide-ranging ornithophilic mosquito vectors such as *Culex quinquefasciatus* [6]. In addition to this wide historic distribution, *Plasmodium* parasites have been introduced to new regions where highly virulent species have led to substantial population declines and extirpations of endemic birds [7]. For example, native avian populations in the Hawaiian Islands and New Zealand have undergone widespread population declines associated with the introduction and spread of avian malaria. Understanding how these introductions have occurred is important to predict how new diseases and vectors of conservation concern will spread and alter other ecosystems and species.

Introduced parasites require all components of their life cycle to thrive in the new ecosystem [8]. For *Plasmodium* to invade a new ecosystem, susceptible bird hosts and a competent mosquito vector are required. There are at least two possible ways avian malaria may have invaded ecosystems: (i) introduction from infected migrating birds and (ii) the release of infected, non-native passerines into the ecosystems. For example, the Bobolink (*Dolichonyx oryzivorus*) in the Galapagos Islands is a *Plasmodium*-carrying passerine that breeds across North America and migrates to central South America, and it is the only passerine with migratory stopovers in the Galapagos [9]. Bobolinks harbor a high diversity of haemosporidian parasites, and analysis of avian malaria parasite lineages across the Galapagos suggests that Bobolinks may have contributed to the spread of avian malaria to these islands because of their migratory behavior [10]. Similarly, in the Hawaiian Islands, shorebirds and other waterfowl are known avian malaria carriers and may have brought these parasites to these islands [11]. Alternatively, avian *Plasmodium* may have arrived in Hawai'i via introduced bird species that carried these parasites and were released into the wild [11]. In the early nineteenth century, several species of nonnative passerine birds were introduced into the Hawaiian ecosystem, many of which were competent carriers of avian malaria parasites. As these introduced species spread, these birds may have also spread the parasites they carried into the wild avian populations [11].

In addition to the introduction of the parasite, the establishment of *Plasmodium* species in new areas requires a competent mosquito vector to sustain the transmission cycle. In Hawai'i specifically, lack of a competent vector likely limited the spread of the *Plasmodium* parasite; although migrating shorebirds and waterfowl likely brought *Plasmodium* species to the islands for thousands of generations, the parasite populations could not have sustained themselves because there were no competent mosquito vectors. However, this was no longer a limiting factor after 1826 when the mosquito *Cx. quinquefasciatus* was unintentionally introduced to the Hawaiian islands along with marine cargo, providing the necessary vector to establish avian malaria *Plasmodium* transmission in the Hawaiian Islands [11].

## Recent advances in understanding avian malaria evolutionary ecology

### Interaction of avian malaria and ecosystem features

Understanding the mechanisms and rates of *Plasmodium* and vector introduction and spread into new ecosystems is critical to prevent population declines and extinctions in immunologically naive bird populations. For example, *Plasmodium* has restricted the range of Hawaiian Honeycreepers. This shrinking distribution is due to *Plasmodium* transmission being limited at higher altitudes where lower temperatures reduce mosquito reproduction and *Plasmodium* development, effectively pushing surviving Honeycreeper populations to higher and higher altitudes [12]. Because of this relationship between mosquito and *Plasmodium* development with temperature, global climate change is likely to further restrict the range of *Plasmodium*-free habitats for many bird species, putting further pressure on these ecosystems. For example, Loiseau et al. [13] demonstrated that *Plasmodium* is transmitted in northern Alaska and also predicted continued range expansion for *Plasmodium* as the area continues to warm. Through these changes, additional populations are expected to become infected with avian malaria and spread *Plasmodium* parasites. Similarly, mechanistic models focused on Hawaiian populations predicted that climate-driven environment and disease patterns will continue to substantially reduce available habitat for native bird populations [14]. Because many Hawaiian passerines are already at high extinction risks due to habitat loss and the introduction of non-native predators, the additional pressure of avian malaria in this and similar systems (e.g. New Zealand and Galápagos Islands) is of high conservation concern [6]. Understanding how to predict the effects of avian malaria and how to limit new vector pathways into additional ecosystems is critical to ongoing conservation action to prevent extinction in these high-risk avian species [14].

### Immune response to avian malaria infection

The survival of avian hosts infected with *Plasmodium* parasites is dependent on the host immune response and its efficiency in detecting and removing *Plasmodium*. One important aspect of adaptive avian immunity is the major histocompatibility complex (MHC). The MHC is essential for survival as it governs the host's ability to detect pathogens, affecting its susceptibility to infections and diseases [15]. The occurrence of specific MHC variants correlates with parasite burden in many Aves species [16], suggesting host genes can influence host fitness by conferring tolerance. In Great Tits (*Parus major*), tolerance to malaria is conferred by two MHC supertypes; individuals with these supertype MHC variants have greater tolerance to malaria compared to individuals without these variants. Importantly, each MHC variant confers tolerance to malaria from a different parasite source (*P. circumflexum* and *P. relictum*) and does so by limiting the physiological effects of infection, not by preventing infection outright [16]. This suggests that susceptibility to avian malaria by the host is dependent on both the virulence of the parasite species and the host’s immunity to the parasite.

Immune responses are also influenced by previous exposures to parasites and diseases. In Canaries (*Serinus canaria*), for example, mortality decreases after reinfection with *Plasmodium* compared to the proportion of individuals that succumb to the first *Plasmodium* infection [17]. Importantly, these effects are not limited to recovery from infection of malaria: *Plasmodium*-infected canaries subjected to a secondary immune challenge are not as effective at eliminating *Plasmodium* compared to those that did not have a secondary challenge. This suggests that there is a tradeoff between control of chronic malaria infection and reaction to new host infections and that this tradeoff may manifest in substantial lifelong effects.

Some immunologically naive populations can evolve resistance and tolerance to avian malaria through natural selection. However, remote island species with reduced genetic variation, such as the Hawaiian species ‘I’iwi (*Drepanis coccinea*) and other species of conservation concern, may lack the genetic diversity or variation in disease response to support adaptation (i.e. no individuals survive infection) [18]. To overcome this, some researchers suggest turning to new gene-editing technologies to bolster the immune system against avian malaria, ultimately helping at-risk populations to recover demographically [18]. For ʻI’iwi, simulated release scenarios suggested that releasing gene-edited ʻI’iwi at mid-elevation forests would substantially reduce extinction risk in the long term [18]. Although this approach has the potential to be feasible and successful, the cultural and ecological implications surrounding modifications of this nature require careful considerations prior to the release of any gene-edited individuals [18]; more research is needed to understand these gene-controlling mechanisms, how introduction of genes can affect wild populations and how these genes will be introduced to wild populations efficiently.

### Incompatible insect technique

Mosquitoes are an essential part of the transmission cycle of avian malaria, as they are the link between infected and uninfected birds. Therefore, one potential way to reduce transmission of avian malaria is to control mosquito populations via chemical means. For example, in New Zealand, the use of insecticides targeting two invasive mosquito species (*Ae. camptorhynchus* and *Cx. sitiens*) led to eradication of these species on the treated island [6]. In contrast, some African populations of *Cx. quinquefasciatus* have evolved resistance to commonly used insecticides, substantially hindering the ability to control mosquito populations [12–21]. The observed concern over the effectiveness of an insecticide-based vector control program has led to an interest in shifting to control by sterile insect techniques (SITs) [22]. Such methods have been successfully used in many insect pest species (e.g. New World screwworm fly, *Cochliomyia hominivorax*; tsetse, *Glossina* spp.) [22]. Males of the target species that are sterilized by radiation or chemicals, genetically modified with lethal genes or harbor incompatible endosymbionts are released into the wild to mate with females, resulting in infertile eggs [23]. Future research to manage and mitigate avian malaria can focus on these and related mechanisms as they provide potential revolutionary mosquito control.

## Future management and mitigation of avian malaria through genetic modifications

Limiting vector populations can occur via several genetic manipulations, including chemosterilization, engineered transgenes, and application of endosymbionts like *Wolbachia* [23–25]. When successful, these modifications are expected to substantially reduce population sizes. However, modifications can also be quickly selected against and removed from the population when the mutations or other genetic changes have large and negative effects on survival [26].

### Using a chemosterilization approach

The use and effectiveness of chemosterilization via a sterile insect technique (SIT) in mosquito control has been extensively tested on *Plasmodium* vectors such as *Cx. quinquefasciatus*. One effective example of SIT deployment in this species has occurred on the island of Seahorse Key; when sterilized male mosquitoes were released onto the island, the island’s larval *Cx. quinquefasciatus* populations were eliminated [27]. However, other attempts to emulate this approach have been less successful because of immigration of mated females from other populations that bypass the sterile male issue [28]. As a result of this spotty effectiveness, new techniques for generating sterile insects may be required, including radiation-based efforts that have been trialed in the apple moth (*Teia anartoides*) in New Zealand [29]. In addition, environmental concerns, mating competitiveness and political climate that has limited the widespread use of sterile insect approaches will have to be overcome before these methods can be deployed widely [6].

### Using genetic engineering systems

The CRISPR-Cas genetic engineering system, which targets specific sequences and results in functional genetic change, has shown great potential in limiting mosquito vector population sizes. In mosquitos, several genes have been identified that would be a suitable gene target for the CRISPR system. These systems work by identifying a homing region where a cargo gene that reduces carrier fitness can ultimately be inserted [30]. As these engineered genes spread through a population, the population declines because of reduced fitness [6]. Importantly, these genetic changes can be engineered to be self-limiting; in the Oxitech^™^ system, female offspring resulting from mating between a male with the cargo gene and a wild female do not survive, providing important safeguards to the modified mosquito system [31]. While this genetic engineering system has merit, use of these technologies requires additional development of safety and regulatory measures. For example, the use of genetic control systems can have negative impacts on the endemic New Zealand *Culex* species, limiting the ways the system could be applied in New Zealand [6]. Using this strategy will require advances for limiting unwanted consequences and conducting in situ tests to determine modification efficiency [32].

### Using endosymbionts

Endosymbionts like *Wolbachia* can also be used to control certain mosquito populations and reduce the occurrence of avian malaria. In some species, when *Wolbachia*-carrying male mosquitoes were released and mated with wild females, the resulting eggs did not hatch and population size was reduced [6]. This approach to limiting mosquito population size has been successful in *Ae. aegypti* in several locations, including the west coast of North America [33]. *Wolbachia* infections have also been deployed as a pathogen-blocking mechanism when infection was not used to prevent egg hatching but instead to block vector parasite infection, diminishing vector competence and disease transmission [34]. However, this approach has its limitations. For example, *Cx. pipens* mosquitos are naturally infected with *Wolbachia* [35], and having a *Wolbachia* infection can increase a mosquito’s susceptibility to *Plasmodium*. This suggests that *Cx. pipens* infected with *Wolbachia* can be better vectors of avian malaria [36]. Thus, the decision to use endosymbiont control methods for mosquito populations requires species-specific information and careful monitoring of mosquito populations. In addition, future work is needed to develop methods for moving and releasing millions of modified mosquitoes to remote locations. Finally, understanding the distribution and diversity of *Wolbachia* and their dynamics with mosquito hosts is important for planning future *Wolbachia*-based control programs [22].

## Conclusions

Avian malaria has caused the decline and extinction of many bird species globally [6]. Since birds are intercontinental migrants, and avian malaria-causing parasites are found worldwide, addressing transmission in the avian malaria system is exceedingly complicated [37]. However, many individuals and species exhibit some resistance or tolerance, suggesting persistence of these species is possible. Targeting the mosquito vector populations may improve health outcomes for many species with mosquito-borne diseases, including humans. However, these vector managing strategies are not without their own risks as each species of mosquito and parasite differs in their relationship to control molecules and their effects on disease spread. Future work is needed in this innovative space on vector control approaches that consider parasite-host eco-evolutionary processes, and research that could help control mosquito vectors of disease relevant to conservation and public health. A downloadable poster describing the the avian malaria life cycle and potential avenues for control is available in Additional file 1: Poster S1."

## Supplementary Information

**Additional file 1: Poster S1.** Avian malaria life cycle and potential avenues for control.

## Data Availability

Not applicable.
